# Supplementary material for: Purification of HCC-specific extracellular vesicles on nanosubstrates for early HCC detection by digital scoring
Source: Nat Commun. 2020 Sep 7;11:4489. doi: 10.1038/s41467-020-18311-0 (PMC7477161; doi:10.1038/s41467-020-18311-0)
Supplement: Supplementary file 1 — Supplementary Information [file 41467_2020_18311_MOESM1_ESM.pdf]

Supplementary Information for

Purification of HCC-specific extracellular vesicles on nanosubstrates for early HCC detection by digital scoring

Na Sun *et al*.

*Correspondence to: hrtseng@mednet.ucla.edu (H.-R.T.); vagopian@mednet.ucla.edu (V.G.A.); rjpei2011@sinano.ac.cn (R.P.); yazhenzhu@mednet.ucla.edu (Y.Z.).

The following sections include:

Supplementary Methods

Supplementary Notes 1,2

Supplementary Figures 1 to 10

Supplementary Tables 1 to 9

Supplementary Methods

Fabrication of polydimethylsiloxane (PDMS) chaotic mixers

PDMS chaotic mixers were fabricated from a photolithographically-prepared master wafer prepared by inductively coupled plasma-reactive ion etching (ICP-RIE). To fabricate the master wafer, a protective layer of chromium and positive photoresist was first cast on top of a 4-inch silicon wafer; the silicon wafer was then exposed to UV light through a photomask of three rectangular microfluidic channels (60 mm in total length and 2 mm in width). The structure of the three microfluidic channels is shown in **Supplementary Figure 1b.** Next, the exposed regions of the photoresist were dissolved away and the exposed chromium was removed with an acid wash. ICP-RIE was then applied to etch the silicon to a depth of 115 µm. A similar procedure was then used to etch 40 µm deep herringbone ridges on top of the silicon wafer. The structure of the herringbone ridges is shown in **Supplementary Figure 1c.** Prior to making replicas via injection moulding, the Si master was pre-treated by exposure to trimethylchlorosilane vapor for 1 min. Well-mixed PDMS precursor (RTV 615 A and B in a 10 to 1 ratio, GE Silicones) was injected to the mold and then incubated in an oven at 80 °C for 48 h to make a 5 mm-thick slab. The resulting PDMS chaotic mixers were peeled off from the silicon master wafer/molds. During injection moulding, two holes were also fabricated at the ends of the channel for insertion of tubing.

Computational simulation and scanning electron microscopy (SEM) for extracellular vesicle (EV) distribution analysis

The PDMS chaotic mixer of the EV Click Chip (**Supplementary Figure 3a**) has three parallel rectangular microfluidic channels (60 mm in total length and 2 mm width) connected head-to-tail. Side-by-side herringbone structures are located on the roof of each rectangular microfluidic channel, with dimensions detailed in **Supplementary Figure 3b**. In short, the channel height (C_H) is 70 µm, herringbone angle (θ) is 90°, herringbone height (H_H) is 40 µm, herringbone length (H_L) is 80 µm, and herringbone width 1 (H_{W1}) and width 2 (H_{W2}) are 80 µm and 160 µm, respectively.

The herringbone structure is capable of passively inducing a microvortex that stirs the flow, facilitating repeated physical contact between Si nanowire substrate (SiNWS) and the flow-through hepatocellular carcinoma (HCC) EVs, further enhancing EV capture performance. Computational fluid dynamics (CFD) was used to study the trajectories of the flow-through HCC EVs in the chaotic mixer. **Supplementary Figure 3c** and **d** show the trajectories of EVs under the assumption that i) the inlet flowrate is 1 mL h^{-1}, ii) the fluid density is 1060 kg m^{-3}, and iii) the viscosity is 0.0036 kg m^{-1} s^{-1}. Near the tops of the nanowires, the velocity of the flow approximates to zero because of the boundary layer and the no-slip condition, allowing the EVs to touch the SiNWS.

Supplementary Figure 3e shows a cross-sectional SEM image of SiNWS with HepG2 EVs captured onto both the tops of the Si nanowires and at different depths (0-1 μm, 1-2 μm, 4-5 μm, and 7-10 μm) along the sidewalls of the Si nanowires. Most HepG2 EVs were captured on the tops of the Si nanowires. Because the flow velocity approximates to zero near the SiNWS in the 1.3 μm thick boundary layer, EVs in this area can diffuse into the SiNWS for click chemistry-mediated capture via Brownian motion. The dissipative particle dynamics (DPD) simulation can predict the balance of forces on the bead (the simulated EV) quickly using Newton's momentum equation shown in equation (1).

$$fi = \sum_{j \neq i} (F_{ij}^{C} + F_{ij}^{D} + F_{ij}^{R}) \quad (1)$$

Where fi is the net force of bead i, bead j is the nearest bead to bead i, F_{ij}^{C} is conservative force, F_{ij}^{D} is dissipative force, and F_{ij}^{R} is random force.

In our DPD simulation, the diameter of EVs was 50 nm, the length, diameter, and spacing of Si nanowires were 10 μm, 100 nm, and 150 nm, respectively. There were a total of 48 EVs in this simulation model. The initial condition placed all EVs above the SiNWS in 2 μm.

Supplementary Figure 3f and **h** show the distribution probability profiles along the depths of the Si nanowires in SiNWS. The results show that the trend from the DPD simulation (n = 48) is similar to the experimental data (n = 108, counted in the SEM micrographs shown in **Supplementary Fig. 3e**), with the distribution probability profile of the DPD simulation showing 37.5%, 15% and 2% of EVs located at depths of the 0 μm (top), 1-2 μm, and 5-6 μm, respectively. **Supplementary Figure 3g** shows the DPD-simulated distribution of the EVs (blue) captured along Si nanowires (brown, depth = 0-2 μm). Most EVs are immobilized on the tops of the Si nanowires.

In our study, the TCO-conjugated antibody agents were incubated with EVs in the artificial plasma samples before being subjected to the EV click chips. The EV recovery yield is flow-rate dependent as shown in **Figure 2g** in the manuscript. To elucidate how the cooperation of the chaotic mixer and SiNWS facilitates the click chemistry-mediated EV capture, we referred to these computational simulation results (**Supplementary Figure 3**) based on computational fluid dynamics (CFD) and dissipative particle dynamics (DPD) models. First, the CFD model simulated the trajectories (**Supplementary Figure 3c/d**) of the flow-through EVs introduced by the embedded herringbone patterns in the chaotic mixer, suggesting that EVs can be effectively introduced into <10 μm-thick boundary layers on SiNWS, where the EVs can then diffuse into SiNWS via Brownian motion for click chemistry-mediated EV capture. The DPD model was then employed to depict Brownian motion of EVs into the Si nanowires on SiNWS to simulate their vertical distribution (**Supplementary Figure 3f-right** and **h**) along the Si nanowires. The simulated results show that the DPD simulated (n = 48) EV distribution is consistent with our optimized experimental data (n = 108) as shown in **Supplementary Figure 3e**, **f-left**, and **g**. According to our simulation model, when flow rate is high (> 2 mL h^{-1}), EVs exhibit limited time in the boundary layers on SiNWS. As a result, there is insufficient time for EVs to diffuse into SiNWS (via Brownian motion) to achieve the desired performance of click chemistry-mediated EV capture.

Calculation of the copy numbers of *C1orf101* and *SRY* transcripts in HCC cell line-derived EVs and background EVs from artificial samples

Artificial plasma samples were prepared by spiking a) 10-µL aliquoted HepG2 cell-derived EVs into 90-µL plasma from a female healthy donor or female cirrhotic patient, b) 10-µL aliquoted SNU387 cell-derived EVs into 90-µL plasma from a male healthy donor or male cirrhotic patient and c) 10-µL aliquoted Hep 3B cell-derived EVs into 90-µL plasma from a male healthy donor or male cirrhotic patient. The EV recovery yield of the male HCC cell line (HepG2) observed for EV Click Chips can be obtained from the following equation (2) (the copy numbers of *SRY* transcripts in the original 10-µL aliquoted HepG2 EVs and the EV Click Chip-recovered HepG2 EVs were denoted as *SRY* $\text{transcripts}_{\text{ori-EV}}$ and *SRY* $\text{transcripts}_{\text{rec-EV}}$, respectively):

$$\text{Recovery Yield} = \frac{SRY\ \text{transcripts}_{\text{rec-EV}}}{SRY\ \text{transcripts}_{\text{ori-EV}}} \qquad (2)$$

The purities of the male HCC cell line (HepG2) EVs harvested from EV Click Chips were calculated as the ratio of recovered *SRY* transcripts (contributed by recovered HepG2 EVs only) to *C1orf101* transcripts (contributed by both recovered HepG2 EVs and the non-specifically captured background female plasma-derived EVs, denoted as *C1orf101*$\text{transcripts}_{\text{rec-EV}}$) using the following equation (3):

$$\text{Recovery Purity} = \frac{n \times SRY\ \text{transcripts}_{\text{rec-EV}}}{C1orf101\ \text{transcripts}_{\text{rec-EV}}} \qquad (3)$$

*n=1.95 is specific to HepG2 EVs.

For HCC cell lines without *SRY* transcripts (SNU 387, Hep3B), cancer cell-derived EVs were spiked into the plasma from a male donor or male cirrhotic patient, and the EV recovery yields and purities can be calculated using the following equations (4) and (5):

$$\text{Recovery Yield} = \frac{C1orf101\ \text{transcripts}_{\text{rec-EV}} - n \times SRY\ \text{transcripts}_{\text{rec-EV}}}{C1orf101\ \text{transcripts}_{\text{ori-EV}}} \qquad (4)$$

$$\text{Recovery Purity} = 1 - \frac{n \times SRY \text{ transcripts}_{\text{rec-EV}}}{C1orf101 \text{ transcripts}_{\text{rec-EV}}} \qquad (5)$$

Characterization of EVs by fluorescence microscopy and dynamic light scattering (DLS)

To track the capture and release processes of HCC EVs spiked in blood plasma samples in EV Click Chips, we conducted two parallel characterization studies. In the first study, RNase-treated HepG2 EVs were first labeled with PKH26 dye and then spiked into healthy donors' plasma samples. The resulting artificial samples were subjected to the HCC EV capture/release workflow shown in **Supplementary Figure 7a**. Again, fluorescent microscopy was employed for tracking the purification (capture/release) process of RNase-treated HepG2 EVs in EV Click Chips. The result is very similar to that observed for HepG2 EVs in PBS (**Figure 3**). In the second study, RNase-treated HepG2 EVs were first exposed to protease K, followed by PKH26 dye labeling. The resulting HepG2 EVs were then spiked into a healthy donors' plasma samples, and these artificial samples were subjected to the HCC EV capture/release workflow shown in **Supplementary Figure 7b**. Fluorescent microscopy imaging revealed neglectable fluorescent signals, suggesting that the click chemistry-mediated capture was not able to immobilize PKH26-labeled HepG2 EVs (with removal of surface proteins) on SiNWS. These results indicated that the enzymatic removal of HCC-associated surface markers on HCC EVs led to the failure of EV capture in EV Click Chips.

The PKH26-labeled EVs were washed with PBS after being immobilized on the chips prior to microscopy imaging to avoid non-specific trapping of free PKH26 dye. Moreover, we conducted a control experiment, where PKH26 dye in healthy donors' plasma (without HCC EVs) was run through the chips. After washing with PBS, no fluorescent signals were observed on the EV Click Chips. Since PKH26 dye can only stain lipid bilayer membrane structured particles, other particles without membranes cannot be stained in this case. These results demonstrated that the fluorescent signals detected under microscopy in our studies can be attributed to labeled EVs rather than non-specific binding of PKH26 micelles or other PKH26 labeled extracellular particles to the nanowires.

Dynamic light scattering (DLS) was adopted to characterize the size distribution of HepG2 EVs in solution. For these studies HepG2 EVs were placed into a disposable microcuvette and analyzed using a Zetasizer Nano instrument (Malvern Instruments Ltd., UK) at room temperature.

Validation of primers and probes for the 10 HCC-specific mRNA markers using ddPCR

To demonstrate the presence of *SRY*, *C1orf101* and the 10 HCC-specific genes, i.e., *alpha-fetoprotein* (*AFP*), *glypican 3* (*GPC3*), *albumin* (*ALB*), *apolipoprotein H* (*APOH*), *fatty acid binding protein 1* (*FABP1*), *fibrinogen beta chain* (*FGB*), *fibrinogen gamma chain* (*FGG*), *alpha 2-HS glycoprotein* (*AHSG*), *retinol binding protein 4* (*RBP4*), and *transferrin* (*TF*), in HCC EVs, we performed the experiments of treating HepG2 EVs and Hep3B EVs with first Protease K followed by RNase to release and remove RNA from ribonucleoprotein complexes before RT-ddPCR. The results summarized in **Supplementary Figure 8b** confirmed the presence of *SRY*, *C1orf101*, and the 10 HCC-specific mRNA markers in HCC EVs. HCC EVs were first incubated with Protease K at 55 ^{o}C for 10 min followed by a treatment for Protease K (Qiagen, German) inactivation at 90 ^{o}C for 10 min according to the manufacturer's protocol. Subsequently, HCC EVs were incubated with RNase at 37 ^{o}C for 30 min followed by RNase inhibitor at 37 ^{o}C for 30 min to remove RNA released from ribonucleoprotein complexes.

To ensure the reproducibility of the ddPCR assay, we validated the PCR primers and probes using cDNA obtained from HepG2 cells, HepG2 EVs, and HCC EVs purified from 5 HCC patients' plasma samples. Each HCC patient's plasma was split into 3 samples for independent analysis for HCC EV purification and HCC-specific mRNA profiling, as shown in **Supplementary Figure 8e,f**.

Supplementary Notes

Supplementary Note 1

All primers and probes were purchased from Thermo Fisher Scientific. We list all their Assay ID and Part Number as followings:

SRY, Hs01026408_cn, 4400291

C1orf101, Hs05767318_cn, 4400291

AFP, Hs01040598_m1, 4448489

GPC3, Hs00170471_m1, 4453320

ALB, Hs00609411_m1, 4448489

APOH, Hs00979406_m1, 4448892

FABP1, Hs00155026_m1, 4448489

FGB, Hs00170586_m1, 4448892

FGG, Hs00241037_m1, 4448489

AHSG, Hs00155659_m1, 4448892

RBP4, Hs00924046_m1, 4448892

TF, Hs00169070_m1, 4448489

Supplementary Note 2

The 10-gene HCC EV Z Score was computed from the RNA expression of the 10 genes using a weighted Z score method in R studio (Version 1.2.1335). The code for generating Z score is shown as following:

```
library(readxl)
expdata <- read_excel("example_data.xlsx")
expmat <- as.data.frame(expdata[,-1])
log2expmat <- log2(expmat+1);
expmed <- apply(log2expmat,2,median)
mc = t(log2expmat) - expmed
std = apply(mc,2,sd,na.rm=TRUE)
zscore <- matrix(0,ncol(mc),1)
for (i in 1:ncol(mc)) {
  zscore[i] = mean(mc[,i],na.rm=TRUE)*sqrt(nrow(mc))/std[i]
}
```

Supplementary Figures

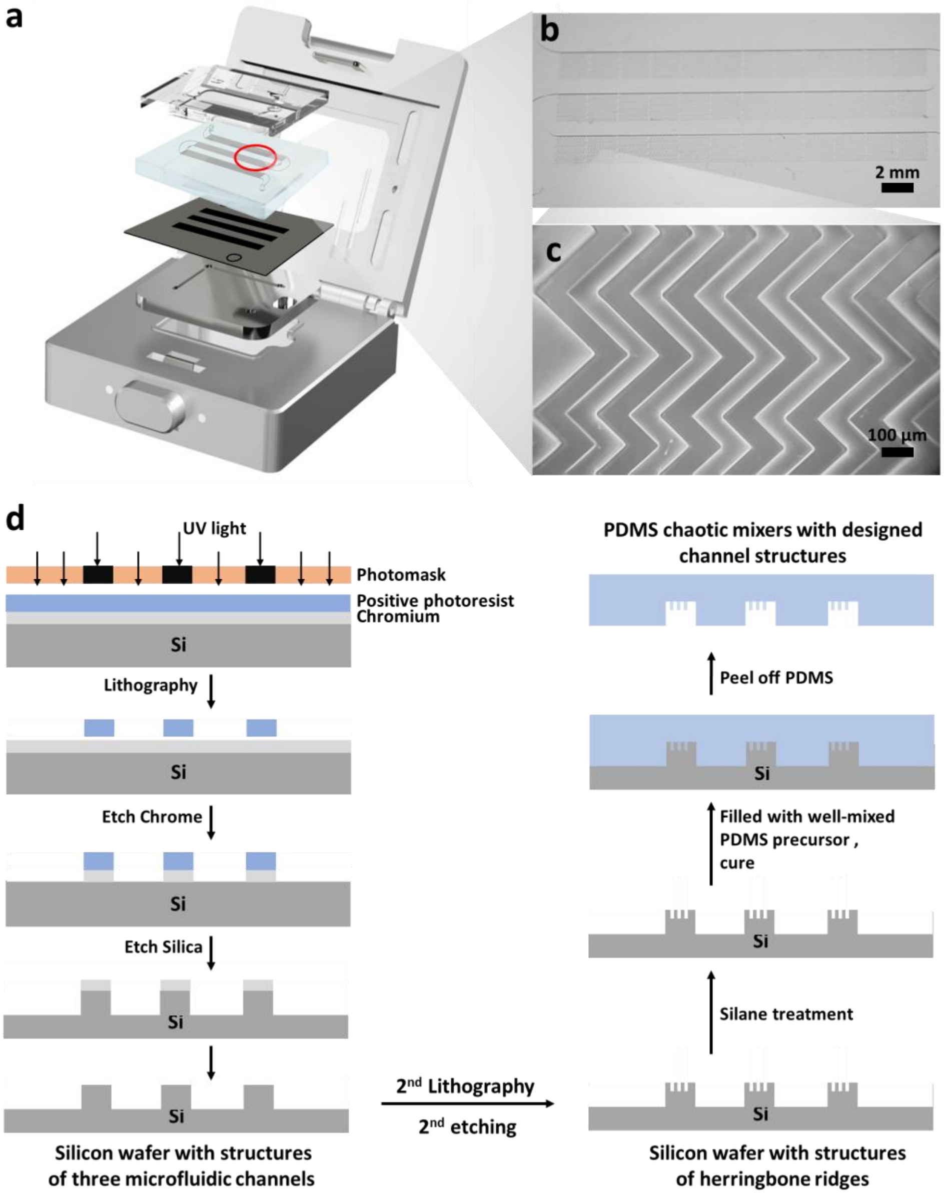

Supplementary Figure 1. Fabrication of polydimethylsiloxane (PDMS) chaotic mixers. (a) A schematic showing the setup of the entire EV Click Chip device. **(b)** A macroscopic picture of three microfluidic channels. **(c)** A microscopic picture of the embedded herringbone patterns on the roof of a PDMS chaotic mixer. **(d)** Workflow of PDMS chaotic mixer fabrication.

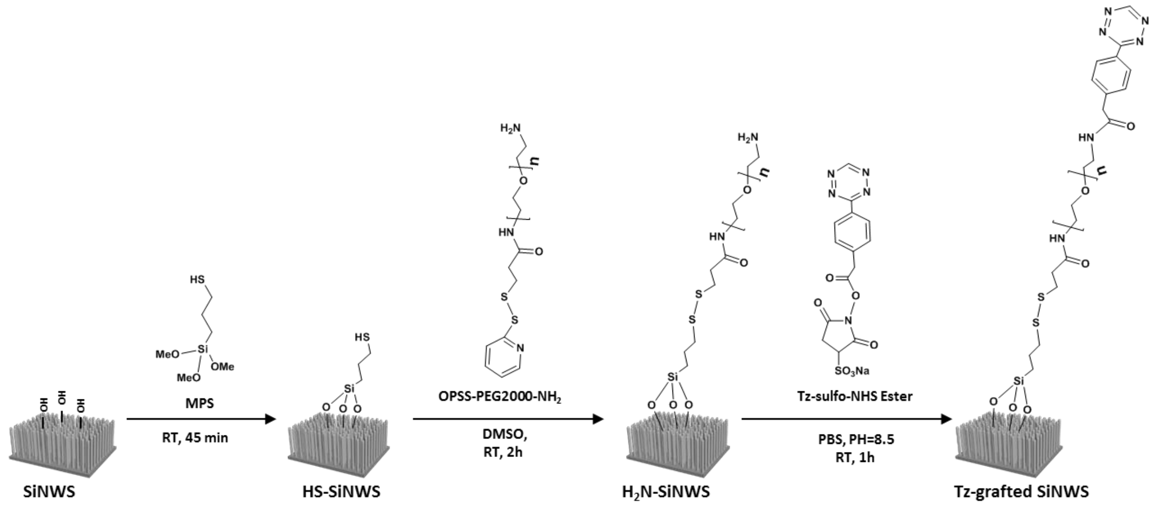

Supplementary Figure 2. Schematic summary of the stepwise functional group transformation employed for the preparation of tetrazine (Tz)-grafted Si nanowire substrate (SiNWS). Tz-grafted SiNWS was prepared via a three-step chemical modification procedure: (i) Silanization: after treating SiNWS with a piranha solution, the resultant SiNWS was treated by (3-mercaptopropyl) trimethoxysilane vapor to give HS-SiNWS; (ii) Incorporation of disulfide bond: HS-SiNWS was reacted with OPSS-PEG-NH_2 in DMSO to introduce disulfide linkers with terminal amine groups (H_2N-SiNWS); (iii) Introduction of Tz motifs: H_2N-SiNWS was treated with Tz-sulfo-NHS ester in PBS solution to generate Tz-grafted SiNWS.

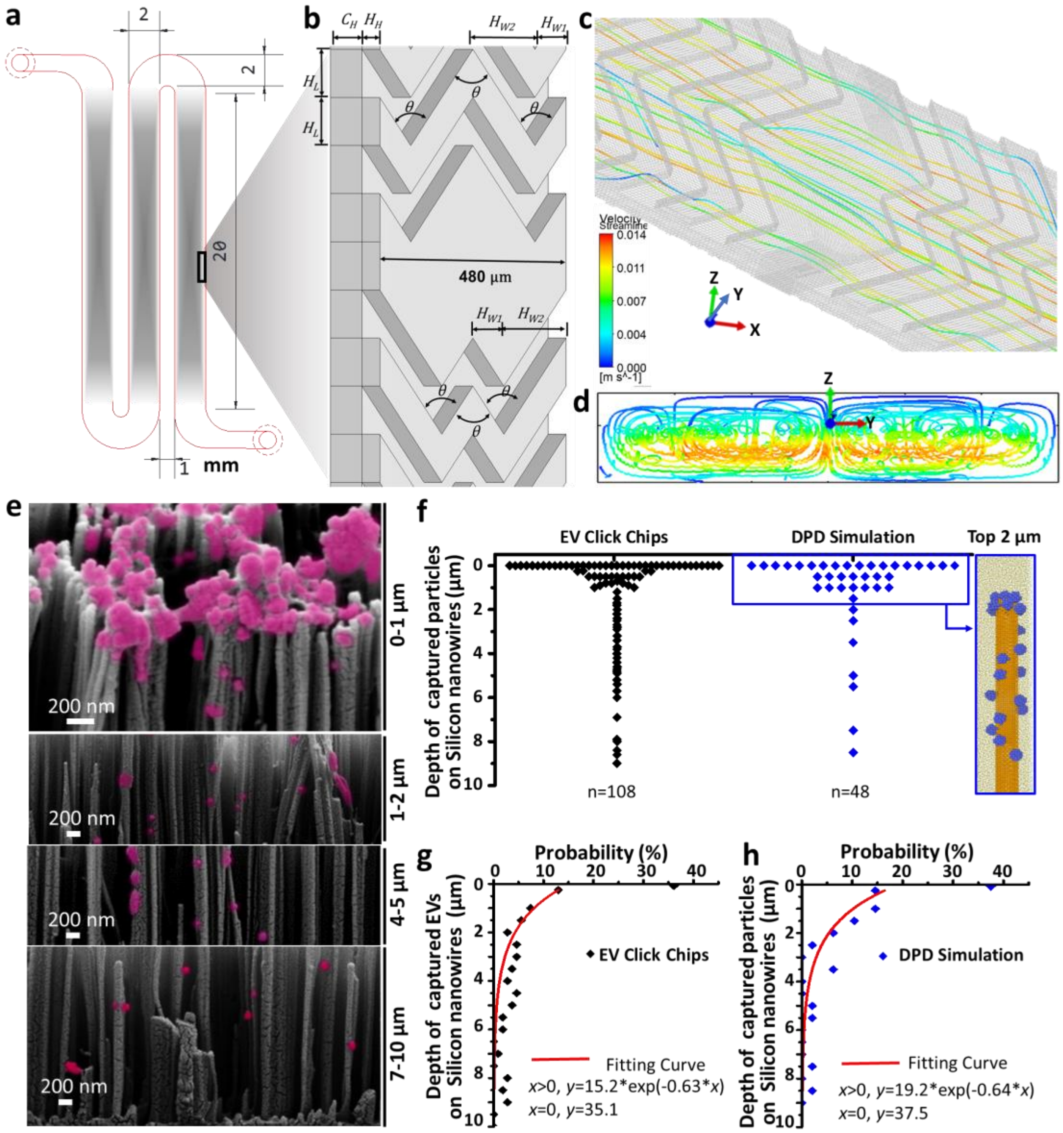

Supplementary Figure 3. Computational simulation and scanning electron microscopy (SEM) for EV distribution analysis. (a) Polydimethylsiloxane (PDMS)-based chaotic mixer channel. **(b)** The geometric configuration of herringbone structures in a PDMS chaotic microchannel. **(c-d)** The simulated trajectories of EVs in the chaotic microchannel. **(e)** SEM image of HepG2 EVs captured onto both the tops of the Si nanowires and at different depths (0-1 μm, 1-2 μm, 4-5 μm, and 7-10 μm) along the sidewalls of the Si nanowires. Data are representatives of three independent assays. **(f-h)** Distribution probability profiles along the depth of Si nanowires. The simulated results show that the dissipative particle dynamics (DPD)-simulated EV distribution (*n* = 48 in the DPD simulation) is consistent with our optimized experimental data (*n* = 108 in the SEM micrographs).

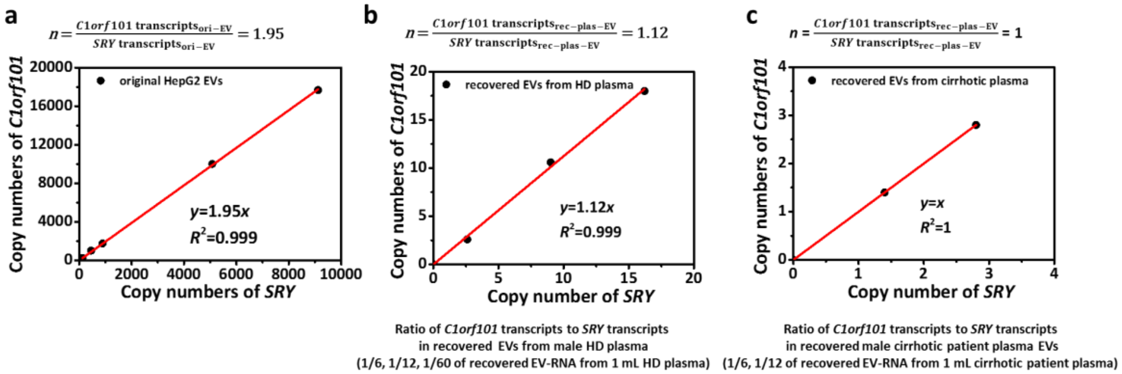

Supplementary Figure 4. Verification of the linear correlation between copy numbers of *C1orf101* and *SRY* transcripts. Linear correlation between copy numbers of *C1orf101* and *SRY* transcripts in (**a**) pure HepG2 EVs, (**b**) background EVs recovered from a male healthy donor plasma sample, and (**c**) background EVs recovered from a male cirrhotic patient plasma sample. HD, healthy donor.

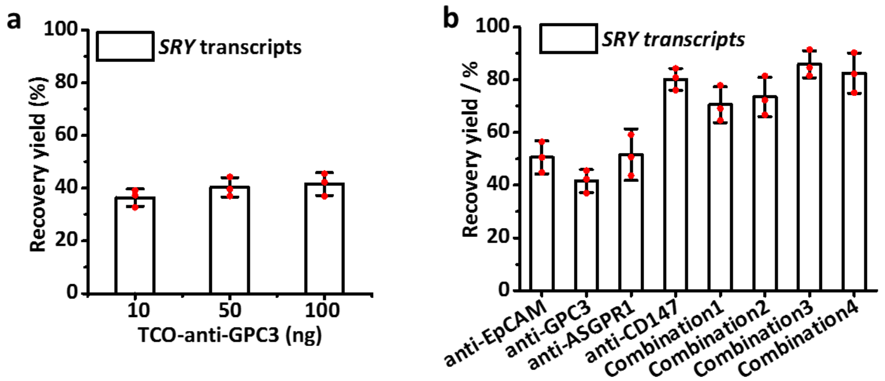

Supplementary Figure 5. Antibody and antibody cocktail optimization and selection. (a) Optimization of the anti-GPC3 concentration for recovering HepG2-derived EVs. Data are means ± SD of three independent assays. **(b)** Comparison of single antibodies and antibody cocktails for recovering HepG2-derived EVs. The HepG2 EV recovery yields were compared among different groups of single antibodies and antibody cocktails (Combination 1: anti-EpCAM & anti-GPC3 & anti-ASGPR1; Combination 2: anti-EpCAM & anti-GPC3 & anti-CD147; Combination 3: anti-EpCAM & anti-ASGPR1 & anti-CD147; Combination 4: anti-EpCAM & anti-GPC3 & anti-ASGPR1 & anti-CD147). Combination 3, i.e., anti-EpCAM & anti-ASGPR1 & anti-CD147, was selected as the optimal multi-marker cocktail for capturing HCC EVs. Data are means ± SD of three independent assays.

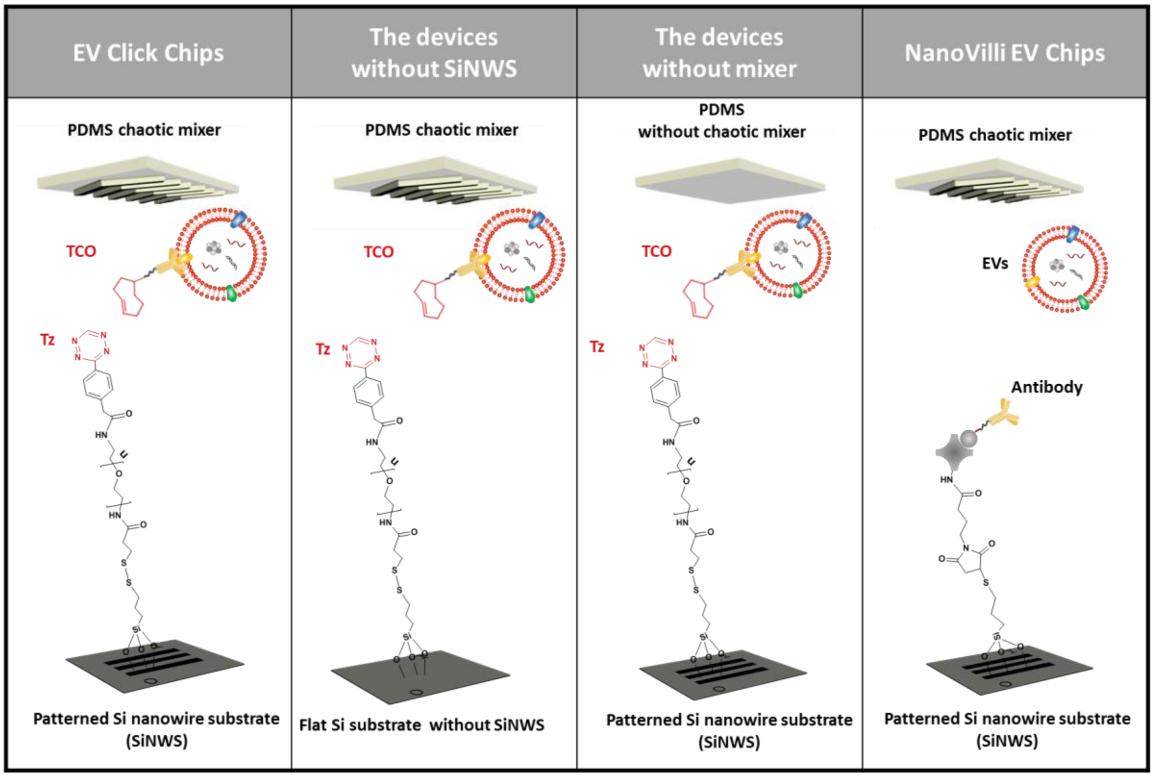

Supplementary Figure 6. Schematic of the EV Click Chip, the control devices without Si nanowire substrate (SiNWS) or polydimethylsiloxane (PDMS) chaotic mixer, and the antibody-antigen mediated capturing system (NanoVilli Chips).

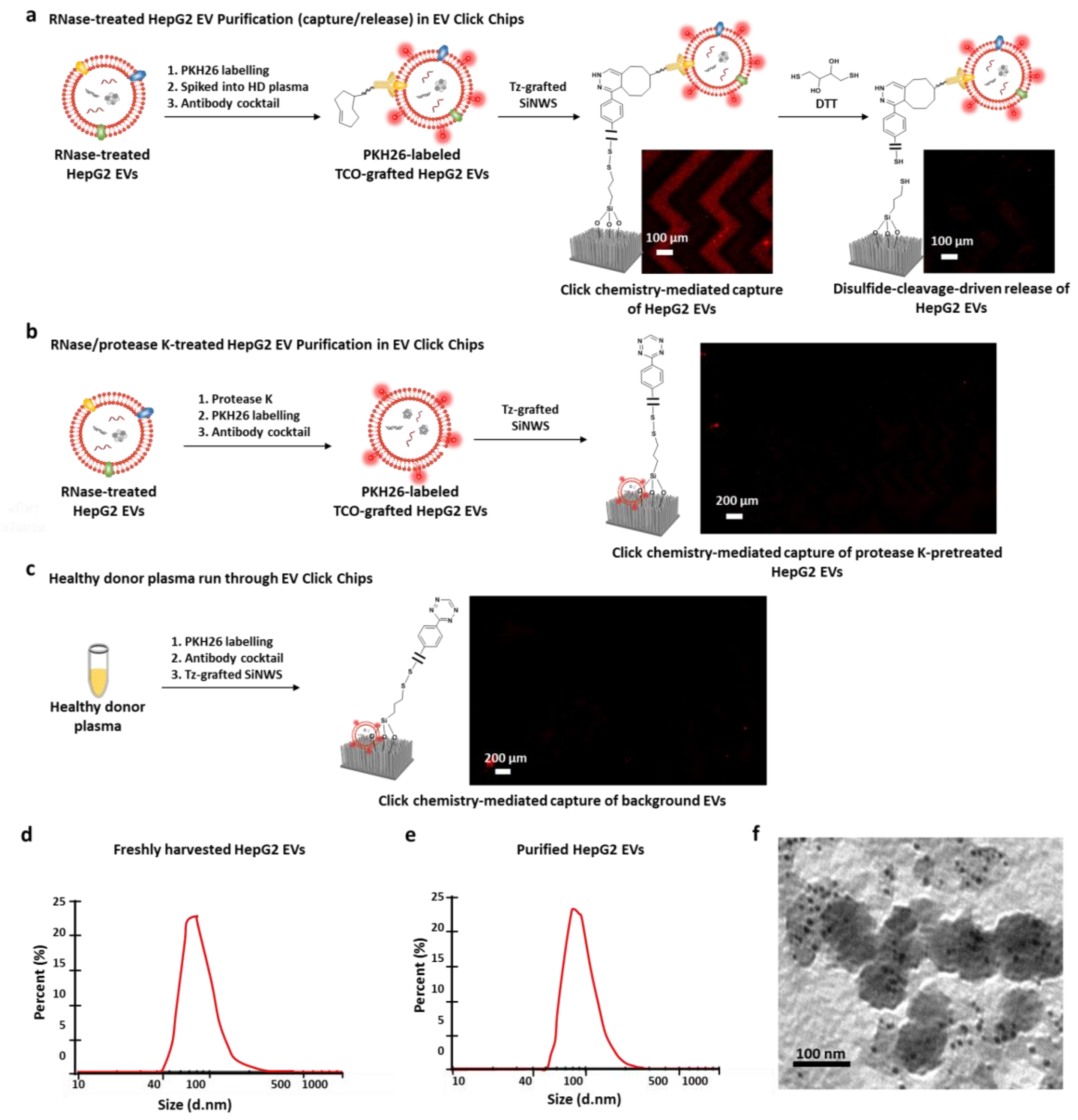

Supplementary Figure 7. **Characterization of HepG2 EVs purified by EV Click Chips.** Tracking the purification (capture/release) process of (**a**) RNase-pretreated HepG2 EVs spiked in healthy donor (HD) plasma and (**b**) RNase-pretreated HepG2 EVs in PBS followed by protease K treatment in EV Click Chips using fluorescent microscopy. (**c**) Tracking the capture process of EVs in healthy donor plasma. Size distribution of HepG2 EVs in solution measured by dynamic light scattering (DLS) (**d**) before and (**e**) after purification by EV Click Chips. (**f**) Immunogold labeling with anti-CD63 for verification of the purified HepG2 EVs from EV Click Chips (10 nm gold particles). For **a**-**f**, data are representatives of three independent assays.

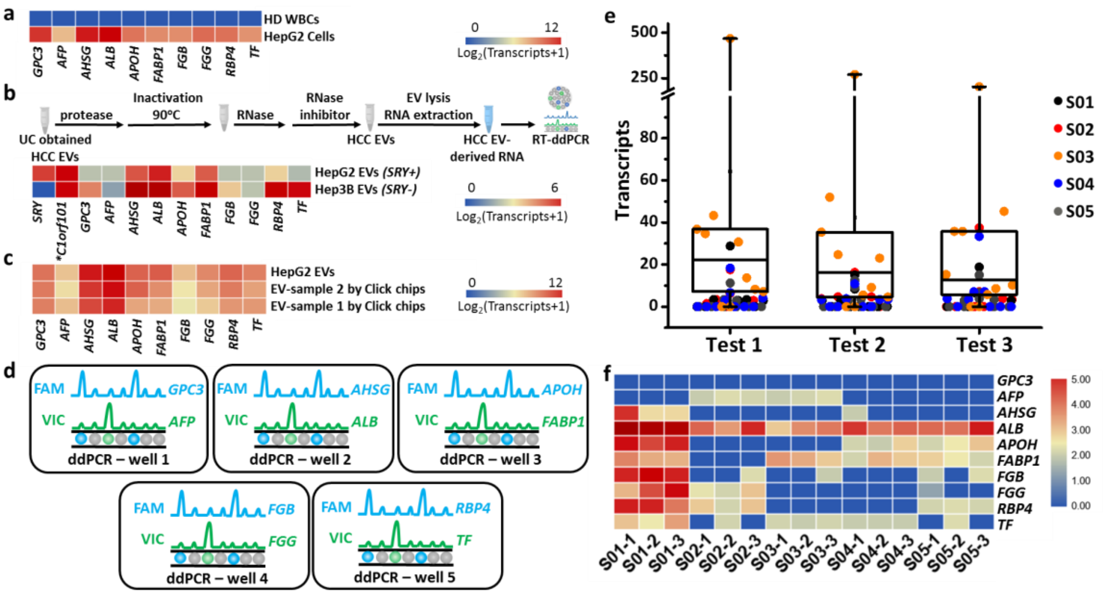

Supplementary Figure 8. Validation of primers and probes for the 10 HCC-specific genes using duplex droplet digital PCR (ddPCR). (**a**) Signals of the 10 HCC-specific genes in HepG2 cells (positive control) and healthy donor (HD) white blood cells (WBCs) (negative control). (**b**) Signals of *SRY*, *C1orf101* and the 10 HCC-specific genes in HepG2 EVs and Hep3B EVs which were treated with Protease K followed by RNase. *Standardized by copy numbers of *C1orf101* transcripts in the 2 cell line-derived EVs. (**c**) Signals of the 10 HCC-specific genes in the original HepG2 EVs, EVs recovered by EV Click Chips using artificial plasma sample (EV-sample 1, HepG2 EVs were spiked into a healthy donor's plasma), and artificial at-risk plasma sample (EV-sample 2, HepG2 EVs were spiked into a liver cirrhotic patient's plasma). (**d**) Schematic illustrating the design and gene assignments in duplex ddPCR for the 10-gene panel. (**e**) The reproducibility study (n = 3 independent tests) of HCC EV-based mRNA assay using 5 HCC patients' samples. Whiskers ranging from minima to maxima, median and 25-75% IQR shown by box plots. Source data are provided as a Source Data file. (**f**) Heatmap of 15 samples from 5 HCC patients' samples for reproducibility study. The intra-class correlation coefficient (ICC) of the 10 HCC-specific mRNA markers for each patient was calculated based upon three repeated measurements (ICC= 0.93; 95% CI, 0.89-0.96).

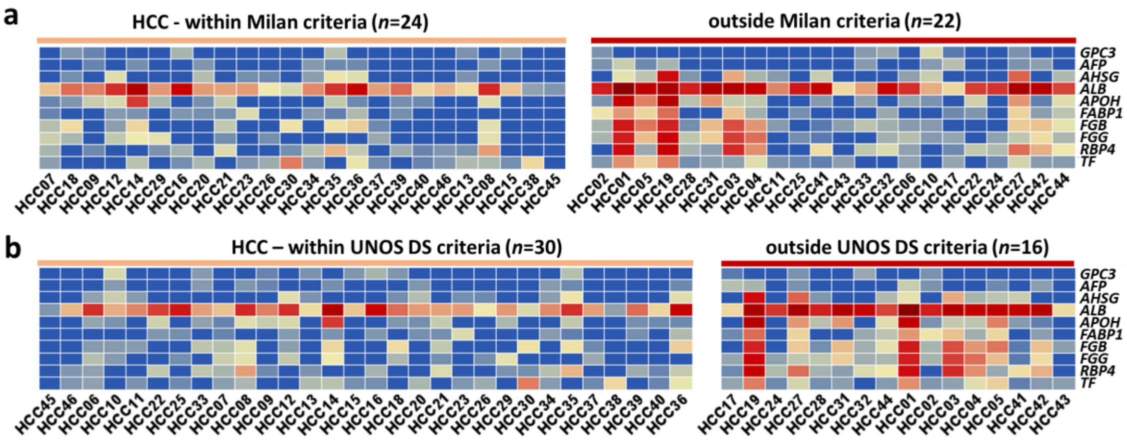

Supplementary Figure 9. Heatmaps of the HCC cohort stratified by (a) Milan criteria and (b) UNOS DS criteria. The signals of the 10 HCC-specific genes of the HCC cohort were summarized in the heat maps according to Milan criteria and United Network for Organ Sharing down-staging (UNOS DS) criteria. Higher signals were observed in advanced-stage (outside Milan, or outside UNOS DS criteria) HCC patients (right), compared with the early-stage (within Milan, or within UNOS DS criteria) HCC patients (left).

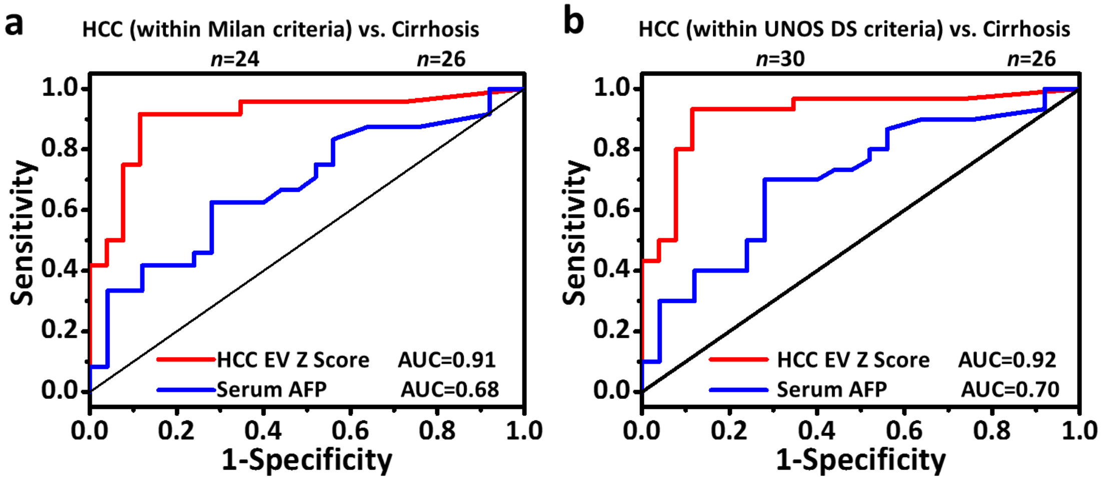

Supplementary Figure 10. ROC analysis for early stage HCC versus cirrhosis. ROC curves comparing HCC EV Z Scores with serum AFP level for differentiating **(a)** HCC within Milan criteria versus at-risk cirrhosis, and **(b)** HCC within UNOS DS criteria versus at-risk cirrhosis. The performances of the HCC EV Z Score and the clinical serum AFP test for differentiating early-stage HCC (within Milan criteria, or within UNOS DS criteria) vs. at-risk liver cirrhosis were compared using ROC analysis. The HCC EV Z Score achieved better diagnostic performance with an AUC of 0.91 and 0.92 in comparison to AFP with an AUC of 0.68 and 0.70 for distinguishing HCC patients within Milan criteria, or within UNOS DS Criteria, from at-risk cirrhotic patients, respectively. AFP, alpha-fetoprotein; AUC, area under the curve; ROC, receiver operator characteristic; UNOS DS criteria, United Network for Organ Sharing down-staging criteria.

Supplementary Tables

Supplementary Table 1. The results of the reproducibility study of *C1orf101/SRY* transcript quantification

Samples	**Ratio of *C1orf101* transcripts to *SRY* transcripts in original cell line EVs (*C1orf101*/*SRY*)**					Intra CV (%)
	Test 1	Test 2	Test 3	Test 4	Mean	
HepG2	2.04 (253/124)	1.97 (1770/894)	1.97 (10020/5080)	1.83 (16680/9120)	1.95	4.51
Male healthy donor 01	1.0 (2.6/2.6)	1.18 (10.6/9)	1.11 (18/16.2)	N/A	1.10	8.27
Male liver cirrhotic patient01	1.0 (2.8/2.8)	1.0 (1.4/1.4)	N/A	N/A	1.0	0

Raw data are shown in table.

Supplementary Table 2. The results of reproducibility study of EV Click Chips using artificial samples

Run Number	**Recovery Yield (%)**				Intra CV (%)
	Test 1	Test 2	Test 3	Mean	
1	73.3	90	93.7	85.7	12.65
2	95.4	81.8	93.8	90.3	8.22
3	87.5	93.8	80.2	87.2	7.83
4	88.5	93.9	80.1	87.5	7.91
5	81.6	81.8	80.1	81.2	1.12
		Inter CV (%)	3.88		

The reproducibility of EV Click Chips was evaluated by calculating the percent coefficient of variation (%CV) for recovery yields. Intra-assay variability was measured for one operator who performed three tests on one day, whereas inter-assay variability was measured across three operators who performed five assay runs total (one run per day), with each run consisting of three tests (15 chips total). Source data are provided as a Source Data file.

Supplementary Table 3. The results of the reproducibility study for the quantitative method for assessing the performance of EV Click Chips using artificial samples of different cell line-derived EVs spiked into different background plasmas

**without RNase**	**Recovery Yield (%)**						**Recovery Purity (%)**					
	Test1	Test2	Test3	Mean	SD	%CV	Test1	Test2	Test3	Mean	SD	%CV
HepG2/HD	82.4	82.4	84.3	83.0	1.1	1.29	91.3	83.7	89.6	88.2	3.3	2.66
SNU387/HD	88.9	88.7	90.8	89.5	1.1	1.33	99.1	98.8	99.1	99.0	0.1	0.14
Hep3B/HD	94.6	90.6	92.3	92.5	2.0	2.17	97.6	96.5	95.2	96.4	1.0	1.02
HepG2/LCD	87.1	96.5	91.8	91.8	4.7	0.78	96.5	89.8	92.9	93.1	2.8	3.99
SNU387/LCD	82.3	81.0	83.4	82.2	1.2	1.46	99.9	98.8	99.9	99.5	0.5	0.47
Hep3B/LCD	81.2	84.2	81.5	82.3	1.7	2.01	92.4	97.5	92.8	94.2	2.3	2.46
	**Recovery Yield (%)**						**Recovery Purity (%)**					
**with RNase**	Test1	Test2	Test3	Mean	SD	%CV	Test1	Test2	Test3	Mean	SD	%CV
HepG2/HD	81.3	85.8	82.3	83.1	2.4	0.78	83.7	86.3	87.9	86.0	1.7	0.64
SNU387/HD	78.7	83.5	83.1	81.8	2.7	3.18	99.7	98.3	97.7	98.6	0.9	0.85
Hep3B/HD	90.6	89.5	83.1	87.7	4.1	6.65	97.9	96.7	97.7	97.5	0.5	0.54
HepG2/LCD	92.9	97.5	90.2	93.5	3.7	1.49	82.8	83.7	81.2	82.6	1.0	1.23
SNU387/LCD	69.5	77.4	76.3	74.4	4.2	5.75	99.5	99.7	98.2	99.1	0.7	0.67
Hep3B/CD	75.4	87.3	74.0	78.9	7.3	9.26	93.0	94.3	87.1	91.5	3.1	3.43

The reproducibility of EV Click Chips was evaluated by calculating the percent coefficient of variation (%CV) for recovery yields and recovery purities. HD, healthy donor; LCD, liver cirrhotic donor. Source data are provided as a Source Data file.

Supplementary Table 4. Clinical characteristics of the HCC cohort

Characteristics	*n* = 46
Age, median (IQR)	67.5 (62-73)
Male, *n* (%)	33 (71.7%)
Race, *n* (%)	
Asian	17 (37%)
African American	5 (10.8%)
Caucasian	17 (37%)
Hispanic	6 (13%)
Unknown	1 (2.2%)
Cirrhosis, *n* (%)	45 (97.8%)
HCC etiology, *n* (%)	
HBV	9 (19.6%)
HCV	18 (39.1%)
ALD	2 (4.4%)
NASH	12 (26%)
Others	5 (10.9%)
Tumor number, *n* (%)	
Single	35 (76%)
Multiple	11 (24%)
Tumor size, *n* (%)	
< 2 cm	2 (4.4%)
2-3 cm	14 (30.4%)
> 3 cm	30 (65.2%)
BCLC stage, *n* (%)	
Stage 0	2 (4.4%)
Stage A	34 (73.9%)
Stage B	2 (4.4%)
Stage C	8 (17.3%)
AJCC stage, *n* (%)	
Stage IA-IB	36 (78.3%)
Stage II	1 (2.2%)
Stage IIIA-IIIB	6 (13%)
Stage IVA-IVB	3 (6.5%)
Milan criteria, *n* (%)	
Within Milan criteria	24 (52.2%)
Outside Milan criteria	22 (47.8%)
UNOS-DS criteria, *n* (%)	
Within UNOS-DS criteria	30 (65.2%)
Outside UNOS-DS criteria	16 (34.8%)
AFP, ng mL^{-1}, median (IQR)	7.6 (3.7-21.3)

AFP, alpha-fetoprotein; ALD, alcoholic liver disease; AJCC, American Joint Committee on Cancer; BCLC, Barcelona clinic liver cancer stage; HBV, hepatitis B virus; HCC, hepatocellular carcinoma; HCV, hepatitis C virus; IQR, interquartile range, NASH, non-alcoholic steatohepatitis; UNOS-DS, United Network for Organ Sharing down-staging.

Supplementary Table 5. Clinical characteristics of the patients with liver cirrhosis

Characteristic	*n* = 26
Age, median (IQR)	62 (54-74)
Male, *n* (%)	19 (73.1%)
Race, *n* (%)	
Asian	5 (19.2%)
African American	2 (7.7%)
Caucasian	11 (42.3%)
Hispanic	5 (19.2)
Unknown	3 (11.5)
Cirrhosis, *n* (%)	26 (100%)
Etiologies, *n* (%)	
HBV	2 (7.7%)
HCV	7 (26.9%)
ALD	11 (42.3%)
NASH	6 (23.1%)
AFP, ng mL^{-1}, median (IQR)	3.3 (2.1-5.7)

AFP, alpha-fetoprotein; ALD, alcoholic liver disease; HBV, hepatitis B virus; HCV, hepatitis C virus; IQR, interquartile range; NASH, non-alcoholic steatohepatitis.

Supplementary Table 6. Clinical characteristics of the patients with chronic hepatitis without liver cirrhosis

Characteristic	*n* = 25
Age, median (IQR)	57 (44-68)
Male, *n* (%)	14 (56%)
Race, *n* (%)	
Asian	18 (72%)
African American	2 (8%)
Caucasian	2 (8%)
Hispanic	1 (4%)
Unknown	2 (8%)
Cirrhosis, *n* (%)	0 (0%)
HBV, *n* (%)	23 (92%)
Viral load, IU mL^{-1}, median (IQR)	11 (0-1921.5)
HCV, *n* (%)	2 (8%)
Viral load, IU mL^{-1}, median (IQR)	N/D
AFP, ng mL^{-1}, median (IQR)	2.2 (1.9-3.5)

AFP, alpha-fetoprotein; IQR, interquartile range; HBV, hepatitis B virus; HCV, hepatitis C virus; N/D, no data.

Supplementary Table 7. Clinical characteristics of the healthy donors

Characteristic	*n* = 23
Age, median (IQR)	53 (38-62)
Male, *n* (%)	14 (61%)
Race, *n* (%)	
Asian	15 (65.2%)
Caucasian	8 (34.8%)

IQR, interquartile range.

Supplementary Table 8. Clinical characteristics of the patients with cancers other than HCC

Characteristic	Metastasis to liver (*n* = 12)	W/o metastasis to liver (*n* = 26)
Age, median (IQR)	61 (52.5-74)	55.5 (50-65)
Male, *n* (%)	12 (100%)	13 (50%)
Cancer type, *n* (%)		
Intrahepatic cholangiocarcinoma	5 (41.7%)	5 (19.2%)
Prostate cancer	0	6 (23.1%)
Neuroendocrine tumor	6 (50%)	2 (7.7%)
Breast cancer	0	7 (26.9%)
Lung cancer	0	6 (23.1%)
Unknown	1 (8.3%)	0

W/o, without; IQR, interquartile range.

Supplementary Table 9. RNA concentrations of patients' plasma samples used in the following RT-ddPCR analysis

Sample ID	RNA concentration (ng μL^{-1})
HCC01	2.63
HCC03	0.53
HCC05	12.00
HCC08	5.37
HCC14	1.77
HCC19	1.08
HCC21	0.10
HCC27	1.96
HCC28	0.42
HCC31	0.04
HCC32	0.19
HCC33	2.25
CLD05	0.82
CLD06	3.00
HD02	0.79
