# Supplementary material for: Purification of HCC-specific extracellular vesicles on nanosubstrates for early HCC detection by digital scoring
Source: Nat Commun. 2020 Sep 7;11:4489. doi: 10.1038/s41467-020-18311-0 (PMC7477161; doi:10.1038/s41467-020-18311-0)
Supplement: Supplementary file 3 — Reporting Summary [file 41467_2020_18311_MOESM3_ESM.pdf]

nature research

Corresponding author(s): Hsian-Rong Tseng

Last updated by author(s): Jul 24, 2020

Reporting Summary

Nature Research wishes to improve the reproducibility of the work that we publish. This form provides structure for consistency and transparency in reporting. For further information on Nature Research policies, see Authors & Referees and the Editorial Policy Checklist .

Statistics

For all statistical analyses, confirm that the following items are present in the figure legend, table legend, main text, or Methods section.

n/a	Confirmed	
☐	☒	The exact sample size (*n*) for each experimental group/condition, given as a discrete number and unit of measurement
☐	☒	A statement on whether measurements were taken from distinct samples or whether the same sample was measured repeatedly
☐	☒	The statistical test(s) used AND whether they are one- or two-sided<br>*Only common tests should be described solely by name; describe more complex techniques in the Methods section.*
☐	☒	A description of all covariates tested
☐	☒	A description of any assumptions or corrections, such as tests of normality and adjustment for multiple comparisons
☐	☒	A full description of the statistical parameters including central tendency (e.g. means) or other basic estimates (e.g. regression coefficient) AND variation (e.g. standard deviation) or associated estimates of uncertainty (e.g. confidence intervals)
☐	☒	For null hypothesis testing, the test statistic (e.g. *F*, *t*, *r*) with confidence intervals, effect sizes, degrees of freedom and *P* value noted<br>*Give P values as exact values whenever suitable.*
☒	☐	For Bayesian analysis, information on the choice of priors and Markov chain Monte Carlo settings
☒	☐	For hierarchical and complex designs, identification of the appropriate level for tests and full reporting of outcomes
☒	☐	Estimates of effect sizes (e.g. Cohen's *d*, Pearson's *r*), indicating how they were calculated

Our web collection on statistics for biologists contains articles on many of the points above.

Software and code

Policy information about availability of computer code

Data collection	Droplet Digital PCR data were collected from QuantaSoft software (Bio-Rad, Hercules, CA)
Data analysis	The 10-gene HCC EV Z Score was computed from the RNA expression of the 10 genes using a weighted Z score method in R studio (Version 1.2.1335). The code to generate Z score has been provided in Supplementary Note 2.<br>ROC curve were evaluated using MedCalc software (version 18.2.1).

For manuscripts utilizing custom algorithms or software that are central to the research but not yet described in published literature, software must be made available to editors/reviewers. We strongly encourage code deposition in a community repository (e.g. GitHub). See the Nature Research guidelines for submitting code & software for further information.

Data

Policy information about availability of data

All manuscripts must include a data availability statement. This statement should provide the following information, where applicable:

- Accession codes, unique identifiers, or web links for publicly available datasets
- A list of figures that have associated raw data
- A description of any restrictions on data availability

Raw data of Figures 2c-f, 2h-j, 4b, 5 and Supplementary Figures 5, 8d are provided as a Source Data file. All the other data supporting the findings of this study are available within the article and its supplementary information files and from the corresponding author upon reasonable request.

Field-specific reporting

Please select the one below that is the best fit for your research. If you are not sure, read the appropriate sections before making your selection.

Life sciences study design

All studies must disclose on these points even when the disclosure is negative.

Sample size	A sample of 31 from the early-stage HCC group and 21 from the control group (the ratio of sample sizes in positive/negative groups is 3/2) will achieve 80% power to detect a difference of 0.20 between our HCC EV digital scoring assay and current clinical screening test for HCC early detection, assuming we will achieve an area under the ROC curve (AUC) of 0.90 for our HCC EV digital scoring assay, and a correlation between the assays of 0.5 and a significance level of 0.05 (two-sided).
Data exclusions	No data were excluded.
Replication	Experimental findings were reliably reproduced at 3 times. The reproducibility study (n = 3 independent tests) of HCC EV-based mRNA assay using 5 HCC patients' samples in Supplementary Figure 8 was performed by different person at different time independently.
Randomization	Patient allocation to each of the cohorts was not random and was defined by their disease states.
Blinding	Clinical annotation of all the plasma samples was performed by a clinician blinded to the assay.

Reporting for specific materials, systems and methods

We require information from authors about some types of materials, experimental systems and methods used in many studies. Here, indicate whether each material, system or method listed is relevant to your study. If you are not sure if a list item applies to your research, read the appropriate section before selecting a response.

Materials & experimental systems

n/a	Involved in the study	
☐	☒	Antibodies
☐	☒	Eukaryotic cell lines
☒	☐	Palaeontology
☒	☐	Animals and other organisms
☐	☒	Human research participants
☒	☐	Clinical data

Methods

n/a	Involved in the study	
☒	☐	ChIP-seq
☒	☐	Flow cytometry
☒	☐	MRI-based neuroimaging

Antibodies

Antibodies used	anti-EpCAM (R&D Systems, Minneapolis, MN, Catalog #AF960)<br>anti-CD147 (R&D Systems, Minneapolis, MN, Catalog #AF972)<br>anti-ASGPR1 (LifeSpan BioSciences, Seattle, WA, Catalog #LS-B11124)<br>anti-GPC3(R&D Systems, Minneapolis, MN, Catalog #AF2119)
Validation	Only commercial and validated antibodies have been used. The validation of each primary antibody for the species and application can be found on the following manufacturer websites.<br>anti-EpCAM, https://www.rndsystems.com/cn/products/human-epcam-trop-1-antibody_af960.<br>anti-CD147, https://www.rndsystems.com/products/human-emmprin-cd147-antibody-109403_mab972.<br>anti-ASGPR1, https://www.lsbio.com/antibodies/ihc-plus-asgr1-antibody-asgpr-antibody-aa100-149-ihc-wb-western-ls-b11124/302240?trid=0<br>anti-GPC3, https://www.rndsystems.com/products/human-glypican-3-antibody_af2119.

Eukaryotic cell lines

Policy information about cell lines

Cell line source(s)	All cell lines (HepG2, Hep 3B and SNU 387) are purchased from ATCC.
Authentication	Authentication was performed by analysis of morphology.
Mycoplasma contamination	All cell lines tested negative for mycoplasma contamination.
Commonly misidentified lines<br>(See ICLAC register)	No commonly misidentified lines were used in this study.

Human research participants

Policy information about studies involving human research participants

Population characteristics	The cohorts in this pilot study included patients with HCC, liver cirrhosis, chronic hepatitis B or C without liver cirrhosis, other cancers with or without metastasis to liver, and healthy donors. Detailed characteristics could be found in Methods and Supplementary Tables 4-8.
Recruitment	All the participants in this pilot study were enrolled between October 2016 - October 2019 at Ronald Reagan UCLA Medical Center and Cedars-Sinai Medical Center. All the participants had to be at least 18 years old. The enrolled HCC patients (n = 46) were treatment-naïve across all stages of the disease. HCC patients who had other malignant tumors or severe mental diseases were excluded. The control cohorts consisted of patients with liver cirrhosis (n = 26), chronic hepatitis B/C without liver cirrhosis (n = 25), other cancers with (n = 12) or without metastasis to liver (n = 26), and healthy donors (n = 23).
Ethics oversight	All patients and healthy donors provided written informed consent for this study according to the IRB protocol (IRB #14-000197) at UCLA and the IRB protocol (IRB #00000066) at Cedars-Sinai Medical Center.

Note that full information on the approval of the study protocol must also be provided in the manuscript.
